# Non-traditional biomarkers of eating disorder symptoms among female college students

**Published:** 2016-12-12

**Authors:** Mara Cristina Lofrano-Prado, Wagner Luiz do Prado, Mauro Virgílio Gomes de Barros, Lila Missae Oyama, Michelle Cardel, Sandra Lopes-de-Souza

**Affiliations:** 1 *Institute of Psychology, University of São Paulo, São Paulo, Brazil*; 2 *Department of Human Movement Sciences, Federal University of São Paulo, São Paulo, Brazil*; 3 *School of Physical Education, University of Pernambuco, Recife, Brazil*; 4 *Department of Physiology, Federal University of São Paulo, São Paulo, Brazil*; 5 *School of Medicine, Department of Health Outcomes and Policy, University of Florida, Gainesville, Florida, United States*; 6 *Department of Anatomy, Federal University of Pernambuco, Recife, PE, Brazil*

**Keywords:** eating disorders, bulimia nervosa, anorexia nervosa, students, peptides, interleukin-6, leptin

## Abstract

**Background:** Eating disorders (ED) are often diagnosed at an advanced stage because traditional symptoms related to unhealthy eating habits are poorly recognized. ED may be also associated with non-traditional and objective biomarkers, which could prove an important screening tool to support health care professionals in diagnosing, treating, and ultimately preventing ED.

**Aim:** To investigate the association between non-traditional physiological ED biomarkers and symptoms of ED among female college students.

**Methods:** This study included 113 female college students, aged 18 to 23 years, enrolled in their first semester as a Bachelor of Health Sciences undergrad at public universities in the urban zone of Recife, Brazil. Symptoms of ED were measured by self-report questionnaires. Circulating levels of IL-6, IL-10, leptin, insulin, ghrelin, PYY and adiponectin were assessed using commercial immunoassays.

**Results:** Students with symptoms of an ED exhibited higher values of IL-6 (*p* = 0.03) and leptin (*p* < 0.001) compared to those without symptoms. A positive correlation was found between leptin levels and bulimia nervosa (*r* = 0.42; *p* = 0.00), between leptin levels and binge eating (*r* = 0.38; *p* = 0.00), and between IL-6 concentrations and binge eating (*r* = 0.25; *p* = 0.04). Multiple linear regression analysis with anorexia nervosa, bulimia nervosa and binge eating as dependent variables showed that IL-6 and leptin best explained ED symptoms, even when adjusted for body mass index (BMI).

**Conclusions:** These findings suggest that peripheral peptides, namely leptin and IL-6, are associated with symptoms of ED in female college students. Future studies are needed to determine if there is a causal relationship between these biomarkers and the onset of ED.

**Relevance for patients:** If future longitudinal studies demonstrate causality between the biomarkers assessed here and ED symptoms, these serum makers could be used as screening tool for inappropriate eating behavior. This may in turn improve the early diagnosis, treatment, and, ultimately, the prognosis of patients with ED.

## 1. Introduction

Energy balance regulation is a homeostatic process controlled by an intricate interplay between the central nervous system (CNS) and various peripheral organs [1]. Briefly, peptides released by peripheral tissues (e.g., muscle, liver, adipocyte) interact with specifics areas of the brain such as the hypothalamus, thereby either stimulating or inhibiting neurons to release anorexigenic (POMC/CART) or orexigenic (NPY/AgRP) neuropeptides that influence eating behavior and energy expenditure [2].

Peripheral signals can be divided in two major categories: 1) adiposity signals, which are involved in the long-term regulation of energy balance, and 2) gut hormones, which govern short-term regulation. Leptin and insulin are the main adiposity signals that inform the CNS about the amount of long-term energy stored in the body. In contrast, gut hormones such as ghrelin, peptide YY (PYY), cholecystokinin (CCK) and pancreatic polypeptide deliver acute signals of satiety and hunger, thereby controlling feeding behavior [3]. Beyond these signals, literature suggests that cytokines such as tumor necrosis factor alpha (TNFα), interleukin-6 (IL-6) and interleukin-10 (IL-10) may also control energy balance [4,5,6]. A disturbance in this system may trigger inappropriate eating behavior, potentially presenting clinically as obesity and/or eating disorders (ED).

ED are psychiatric disorders characterized by extreme dietary restraint followed by episodes of binge eating, purgative behavior, and an overvalued idea about weight [7]. The prevalence of ED has increased worldwide since 1930 [7,8]. The prevalence of anorexia nervosa (AN) and bulimia nervosa (BN) among young females is approximately 0.6% in US and 1% in Europe. The prevalence of binge eating in the US population is around 3% [9]. Data from the National Association of Anorexia Nervosa and Associated Disorders [10] demonstrate that 86% of patients have the onset of ED by the age of 20. Health sciences students are at increased risk to present ED [11,12]. It is not clear whether this is a cause of a consequence of their choice of study. Fiat and Salles [11] suggest that people who are worried about personal appearance (e.g., body weight and size) have a personal motivation to study this topic and are therefore more likely to choose health science courses.

Given that ED patients tend to hide classical ED symptoms from their relatives, ED are often not diagnosed until late stages [9] and therefore often go untreated for long periods of time [13,14]. The identification of non-traditional and objective biomarkers associated with symptoms of ED may prove a new tool to prevent and treat ED. Thus, the aim of this study was to investigate the associations between non-traditional ED biomarkers, including IL-6, IL-10, leptin, insulin, ghrelin, PYY and adiponectin and symptoms of ED in female college students.

## 2. Materials and Methods

### 2.1. Participants

This cross-sectional study included women aged 18 to 23, enrolled in their first semester in a bachelor of Health Sciences undergrad program (including Medicine, Nutrition, Physical Education, Physiotherapy, Dentistry, Nursing, and Occupational Therapy) at public universities in the urban zone of Recife, Northeast of Brazil. Exclusion criteria included previous enrollment in other Health Science classes (to avoid bias based on previous knowledge about the instruments), taking anti-inflammatory drugs, pregnancy, self-reported chronic disease, flu, allergy, and/or illness on the day of blood sampling and/or diagnoses or in treatment for ED (self-reported). This study was conducted in accordance with the principles of the declaration of Helsinki and was formally approved by the ethical Institutional Review Board of the Federal University of Pernambuco (CAAE 0143.0.172.000-10). Informed consent was obtained from all subjects.

The sample size was estimated using a 5% margin of error (95% confidence interval [CI]). A design effect of 1.5 and a 50% expected prevalence of ED symptoms was based on previously studies [15,16,17,18]. The estimated sample size was sufficient to detect significant r values greater than or equal to 0.41, with a power of 80% and a 95% CI.

### 2.2. Measurements

During the first visit, the responsible researcher provided information about the study aim and the study procedures and informed consent was obtained. During a next visit, those who agreed to participate filled out questionnaires, anthropometric measurements were done, and blood samples were taken. As is common in Brazil, participants were not compensated for study participation.

#### 2.2.1. Anthropometric measurements

Participants were weighed wearing light clothing and no shoes on a Filizola scale (Model 160/300, Brazil). Weights were rounded to the nearest 0.1 kg. Height was measured using a wall-mounted stadiometer Filizola (Model 160/300, Brazil) and rounded to the nearest 0.5 cm.

#### 2.2.2. Eating behavior symptoms

Self-report questionnaires were filled out in a quiet room before the anthropometric procedures.

Eating Attitudes Test (EAT-26) was translated and validated for the Brazilian population by Nunes et al. [19] and has a good internal consistency, as is reflected by a reported Cronbach alpha rating of 0.75 [20]. EAT-26 is a self-report instrument used to evaluate and identify abnormal eating patterns. The instrument comprises 26 items with six options: always = 3 points, very frequently = 2 points, frequently=l point, sometimes/rarely/never = 0 point. Cumulative scores over 20 points were considered indicative of abnormal eating behavior and an increased risk of developing anorexia nervosa [21].

Bulimic Investigatory Test Edinburgh (BITE) was translated and validated for the Brazilian population by Cordás and Hochgraf [22]. Test-retest reliability for the Brazilian version was reported at a Kappa coefficient of 0.85 [23]. BITE is a 33-item self-report measure, designed to identify subjects with symptoms of bulimia or binge eating. The BITE is divided into two subscales: the symptom scale and the severity scale. All the questions, with the exception of the three starred questions (6, 7 and 27), make up the symptom scale. The underlined questions (1, 13, 21, 23 and 31) score one point for a “no” response. The remaining 25 items score one point for a “yes” response. The maximum score is 30. Scores of the symptom scale can be subdivided into three groups: high scores (higher than 20) medium scorers (between 10 and 19) and low scorers (below 10). The total score is the sum of the answers [24].

Binge Eating Scale (BES) was translated and validated for the Brazilian population by Freitas et al. [25]. The internal consistency of the Brazilian version, reflected by the Cronbach’s alpha coefficient, was 0.89 [26]. BES is a 16-item self-reported questionnaire designed specifically to identify behavioral and cognitive characteristics of binge eating. Each item presents three or four differently weighted statements, with a final score ranging from 0–46. Based on the scores, disturbed eating behavior is classified into three different levels of severity: non-bingers (scoring 17 or less), moderate bingers (scoring between 18 and 26) and severe bingers (scoring 27 or higher) [27].

Individuals who scored above 20 points on EAT and/or above 9 points on BITE and/or above 17 points on BES were considered as showing symptoms of ED (SED). Those scoring below the cut-off points were classified as having no symptoms of ED (NED).

#### 2.2.3. Serum analyses

Blood samples were collected in the outpatient clinic at approximately 8:00 a.m., after an overnight fast (12 hours). Study participants were asked to avoid intense exercise 48 hours prior to sample collection. Serum was separated by centrifugation and stored at –80 °C. Serum insulin, leptin and PYY_3-36_ were measured by enzymatic immunoassays, using commercial enzyme-linked immunosorbent multiplex panel assay kits (assay sensitivity: insulin (44.5 pg/mL), leptin (157.2 pg/mL) and PYY (8.4 pg/mL)). Total ghrelin (assay sensitivity: 100pg/ml) and adiponectin (assay sensitivity –0.78 ng/mL) were assessed using commercial enzyme-linked immunosorbent assays (Millipore Corporation). IL-6 and IL-10 were also measured using enzyme-linked immunosorbent assays (Bender System).

### 2.3. Statistical analysis

All data were analyzed using SPSS version 10 for Windows. Komolgorov-Smirnov test was performed to assess the normality of the residuals. The values of the peripheral peptides were converted to natural logarithms (LOG) to normalize the distribution and are presented as mean (M) ± standard deviation (SD). Comparisons between groups (SED vs NED) were assessed by Student’s t-test for independent groups. Simple and partial correlation analyses (controlling for BMI) between symptoms of eating disorders (anorexia, bulimia and binge eating) and leptin, ghrelin, IL-6, IL-10, adiponectin, PYY and insulin were performed using Pearson’s coefficient test. Forward multiple linear regression having EAT, BITE and BES as dependent factors and peptides as independent variables were performed. Significance was set at p ⩽ 0.05.

## 3. Results

Two hundred eighty-three students volunteered for the study. One hundred six potential participants were excluded from the study because they refused blood work, and another 64 were excluded at the day of blood sampling because they met one or more of the exclusion criteria. No demographic differences between the subjects who refused blood work and the participants who consented were observed. Thus, 113 female college students were included in the analysis, of which 47 (42%; 95%CI = 32.8-50.8%) were classified in the SED group and 66 (58%; 95%CI = 49.2-67.2%) in the NED group. Demographic baseline data are found in Table 1. SED students were heavier (*p* < 0.001) and had a higher BMI (*p* < 0.001) than NED. No significant differences were observed for age and height.

Table 1 compares the circulating levels of peripheral peptides between groups. SED group had higher values of IL-6 (*p* = 0.03) and leptin (*p* < 0.001) compared to NED. No differences between groups were found for IL-10 (*p* = 0.59), adiponectin (*p* = 0.75), ghrelin (*p* = 0.57), PYY (*p* = 0.21) and insulin (*p* = 0.41).

**Table 1:** Anthropometric characteristics of college students with and without symptoms of eating disorders

Variables	SED(*n* = 47)	NED(*n* = 66)
	M ± DP	M ± DP
Age (y)	19.14 ± 1.08	19.64 ± 1.52
Height (m)	1.62 ± 0.06	1.63 ± 0.06
Body Mass (Kg)	61.34 ± 12.98	54.46 ± 8.17*
BMI (Kg/m^2^)	23.06 ± 4.10	20.46 ± 2.77*

SED = symptoms of eating disorders; NED = no symptoms of eating disorders; BMI = Body Mass Index;

*p < 0.001

**Table 2:** Descriptive analyses of biochemical parameters

Variables	Symptoms of ED (*n* = 47)	No symptoms (*n* = 65)
	M ± SD	M ± SD
LOG IL-10	0.078 ± 1.355	0.246 ± 1.446
LOG IL-6	0.500 ± 1.003	-0.073 ± 1.045*
LOG Adiponectin	2.420 ± 0.348	2.444 ± 0.398
LOG Ghrelin	5.946 ± 0.614	6.017 ± 0.685
LOG Leptin	2.691 ± 0.537	2.337 ± 0.582*
LOG PYY	4.205 ± 0.435	4.086 ± 0.508
LOG Insulin	2.196 ± 0.465	2.125 ± 0.422

LOG = natural logarithms; IL-10 = interleukin 10; IL-6 = interleukin 6; PYY = Peptide YY;

*p < 0.05

In the full cohort, a positive correlation between leptin levels and BITE and BES scores, and between IL-6 and BES was observed (Table 1). After correcting for BMI, IL-6 was correlated with EAT (*r* = 0.328, *p* = 0.02) and BITE (*r* = 0.273, *p* = 0.05), and there was a correlation between adiponectin and BES (*r* = 0.275, *p* = 0.05). Multiple linear regression analyses including BITE and BES as dependent variables showed that IL-6 and leptin is the best model to explain the symptoms of BN and binge eating (Table 1), even when adjusted for BMI. Related to EAT, the best model included IL-6 (*r*^2^ = 0.064, *p* = 0.06) and leptin (*r*^2^ = 0.064, *p* = 0.33), even when adjusted for BMI. No associations for ghrelin, IL-10, PYY, adiponectin and insulin were found.

**Table 3:** Simple correlation coefficients between EAT, BITE and BES scores and biochemical parameters in young woman with symptoms of eating disorders

	EAT	BITE		BES		
	r	P	R	P	r	P
LOG IL-10	−0.129	0.27	−0.119	0.86	0.111	0.30
LOG IL-6	0.223	0.07	0.224	0.07	0.247	0.04
LOG Adiponectin	0.149	0.11	−0.009	0.92	0.112	0.24
LOG Ghrelin	−0.006	0.95	−0.038	0.69	0.160	0.09
LOG Leptin	0.103	0.28	0.419	0.00	0.376	0.00
LOG PYY	0.121	0.21	0.114	0.24	−0.010	0.71
LOG Insulin	0.126	0.19	0.085	0.37	0.069	0.47

EAT= Eating Attitudes Test; BITE = Bulimic Investigatory Test Edinburgh; BES = Binge Eating; Scale; LOG = natural logarithms; IL-10 = interleukin 10; IL-6 = interleukin 6; PYY = Peptide YY

**Table 4:** Multiple regression analysis of BITE and BES as dependent variable

	R	R^2^	Adjusted R^2^	Beta	P	95% CI
BITE	0.489	0.239	0.215			
LOG IL-6				0.253	0.02	(0.154, 2.325)
LOG Leptin				0.436	0.00	(1.883, 5.792)
BES	0.465	0.216	0.191			
LOG IL-6				0.273	0.01	(0.275, 2.951)
LOG Leptin				0.395	0.00	(1.743, 6.383)

BITE = Bulimic Investigatory Test Edinburgh; BES = Binge Eating; Scale; LOG = natural logarithms; IL-6 = interleukin

## 4. Discussion

To our knowledge, this is the first study exploring the associations between non-traditional ED biomarkers (IL-6, IL-10, leptin, insulin, ghrelin, PYY and adiponectin) and symptoms of ED in female college students. The main findings are: 1) students with symptoms of ED have higher circulating levels of IL-6 and leptin than students without symptoms, and 2) IL-6 and leptin circulating levels are positively associated with symptoms of AN, BN and binge eating.

It has been hypothesized that peripheral cytokines could play an etiological role in the development of ED. Experimental studies observed that peripherally and centrally, pro-inflammatory cytokines such IL-1, IL-6 and TNFα induce changes in neurochemical, behavioral and physiological parameters. This has also been observed in patients with AN [28]. The results are however controversial and studies for BN are limited [4].

Previous work has demonstrated that women with a diagnosis of AN have lower leptin [29] and insulin levels [30], and higher systemic ghrelin [31] and PYY [32] concentrations compared with their counterparts without AN, reflecting a negative energy balance in these patients. Similarly, Tagami et al. [33] evaluated woman (19-48 years old) with AN and BN and found lower adiponectin concentrations in patients with AN (BMI = 14.0 ± 2.5) and BN (BMI = 20.5 ± 1.8) compared with normal-weight subjects (BMI = 20.3 ± 1.5). Conversely, Karczewska-Kupczewska et al. [30] showed that serum adiponectin levels were higher in AN women. Monteleone et al. [34] compared healthy women (22.6 ± 2.7 years old; BMI = 21.8 ± 1.7) with BN patients (24.3 ± 2.6 years old; BMI = 22.2 ± 3.5) and with binge eating disorder (BED) patients (30.6 ± 9.4 years old; BMI = 33.9 ± 7.0). The latter study showed that BN patients exhibited significantly increased plasma levels of adiponectin, while woman with BED had significantly reduced blood concentrations of adiponectin compared with healthy counterparts.

All previous studies were conducted in patients with a diagnosis of AN (BMI average around 15.00 Kg/m^2^). This differs from our study population, which was composed of female college students, with and without symptoms of an ED, but with no current ED diagnosis. The mixed results observed in the literature could be attributed to the differences in study population. It is however important to note that in both cases there is evidence to support a role for these peptides in the pathophysiology of ED.

Despite the evidence linking gut hormones, adiposity signals and cytokines with ED [35], it remains unclear whether disturbances in peripheral peptides secretion is a cause or consequence of these psychiatric disorders [4,5,36,37]. Based on the results of the present study, we suggest that prior to an official diagnosis of ED, associated physiological changes are already present.

The present study has some limitations that should be considered. ED symptoms were measured with self-report instruments, rather than being diagnosed by a psychologist or psychiatrist. Due the direct association between adiposity and the circulating levels of the measured peptides, the absence of body composition measures is a significant limitation. The lack of information related to chronic diseases associated with inflammatory processes that could alter the levels of the peptides should also be considered when interpreting the current data. Unfortunately, technical and sample availability issues prevent us from assessing TNFα, an important biomarker implicated in the central control of eating behavior, and other inflammatory mediators. The high number of individuals who refused blood work should also be considered.

In conclusion, the present study found a positive association between circulating levels of IL-6 and leptin and symptoms of ED in college students without an ED diagnosis. However, further studies are needed to determine whether there is a causal relationship between these biomarkers and the onset of ED. If the analyses of these non-traditional biomarkers would be included in the routine psychiatric screening for ED, this could have significant implications for earlier clinical detection and treatment of ED. As such it has the potential to improve long-term patient outcomes.
